# Interspecific variation and functional traits of the gut microbiome in spiders from the wild: The largest effort so far

**DOI:** 10.1371/journal.pone.0251790

**Published:** 2021-07-21

**Authors:** Kaomud Tyagi, Inderjeet Tyagi, Vikas Kumar

**Affiliations:** Centre for DNA Taxonomy, Molecular Systematics Division, Zoological Survey of India, Kolkata, India; Babasaheb Bhimrao Ambedkar University, INDIA

## Abstract

Spiders being one of the most diverse group in phylum arthropod are of great importance due to their role as predators, silk producer, and in medicinal applications. Spiders in prey–predator relationships play a crucial role in balancing the food-chain of any ecosystem; therefore it is essential to characterize the gut microbiota of spiders collected from natural environments. In the present work, the largest effort so far has been made to characterize the gut microbiota of 35 spider species belonging to four different families using 16S amplicon targeting sequencing. Further, we compared the gut microbiota composition including endosymbiont abundance in spider species collected from different geographical locations. The results obtained revealed the presence of genera like *Acinetobacter* (15%), V7clade (9%), *Wolbachia* (8%), *Pseudomonas* (5%), *Bacillus* (6%). Although comparative analysis revealed that the gut bacterial composition in all the spider families has a similar pattern, in terms of community richness and evenness. The bacterial diversity in the spider family, Lycosidae are more diverse than in Salticidae, Tetragnathidae and Araneidae. Furthermore, it was observed that the abundance of endosymbiont genera, i.e. *Wolbachia* and *Rickettsia*, leads to shift in the abundance of other bacterial taxa and may cause sexual alterations in spider species. Moreover, predicted functional analysis based on PICRUSt2 reveals that gut microbiota of spider species were involved in functions like metabolism of carbohydrates, cofactors and vitamins, amino acids; biosynthesis of organic compounds, fatty acids, lipids etc. Based on the results obtained, it can be said that different locations do not correlate with community composition of gut microbiota in spider species collected from natural environments.

## Introduction

The functioning and survival of living organisms in an ecosystem are greatly influenced by the gut microbiome they possess [1, 2]. Recent research has shown that gut microbiota plays a significant role in determining mating preference [3], reproductive manipulation [4], inhibiting pathogen transmission [5, 6] and insecticidal resistance [7]. Moreover, gut bacterial diversity is also linked to various physiological processes like digestion, detoxification, or nutrient supplementation [5, 8–10]. The emergence of Next Generation Sequencing (NGS) has revolutionised the study of gut bacterial diversity [11]. While, NGS-based gut bacterial diversity assessment and their functional pathways has been widely studied in many groups of arthropods [12], including insects [8, 9, 13–15], scorpions [16], ticks [17], and termites [18]. So far, few species of spiders have been studied for their gut bacterial diversity [19–23]. The spiders used for the gut microbiome analysis in earlier studies were either reared or diet-driven which play an important role in shifting the gut microbiome composition.

With more than 48,000 described species worldwide, spiders are a diverse group of predators [24] and distributed globally in all the terrestrial ecosystems [25–27]. Spiders are known as “liquid feeders”, as they have extra oral digestion (EOD) [20] and this peculiar way of ingestion potentially affects the microbiome composition in this group. So, the survey of gut bacterial diversity and their predicted metabolic functions in spiders is of pivotal importance due to their medicinal and ecological significance.

In 2010, Wand screened 31 Chinese spider species to understand the horizontal transmission of *Wolbachia* between prey and predator [28]. This study detected the presence of *Wolbachia* in only 7 species (*Eriovixia cavaleriei*, *Larinia argiopiformis*, *Araneus ventricosus*, *Nephila clavata*, *Oxyopes sertatus*, *Pholcus crypticolens* and *Coleosoma octomaculatum*). Later, Vanthournout and colleagues studied the involvement of *Wolbachia*, *Rickettisia*, and *Cardinium* in sex ratio variation in the dwarf spider *Oedothorax gibbosus* [29]. Hu et al. in 2018 detected *Wolbachia* and *Rickettsia* in the spider species *Nurscia albofasciata* [21]. In this study, we made the first large-scale attempt to decipher the community composition of the gut microbiota in natural populations of 35 spider species belonging to four families (Araneidae, Lycosidae, Salticidae, and Tetragnathidae) using the NGS amplicon data. We used statistical methods to compare the gut microbiome of spider species. The present study will enhance our knowledge on the gut microbiome and their interfamilial relationship.

## Results

### Gut bacterial composition among the four spider families

**Fig 1:**
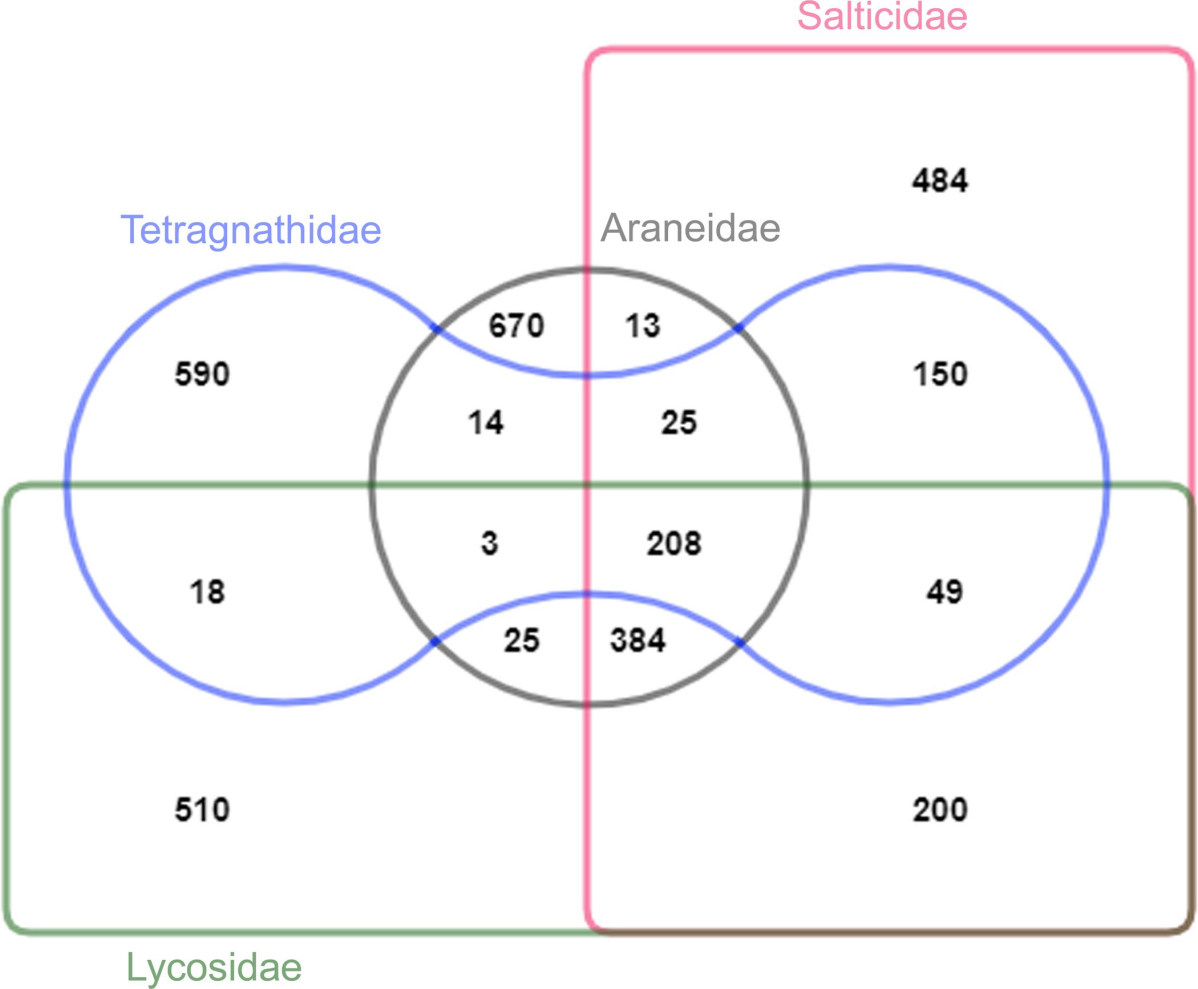
Venn diagram illustrates overlap of ASVs in gut bacterial diversity among the four spider families.

To investigate the composition and diversity of gut microbes in spiders, we included 35 species of spiders belonging to four families, Araneidae (12), Lycosidae (7), Salticidae (10), and Tetragnathidae (7). However, one species, *Thiania bhamoensis* of the Salticidae family, was sequenced from two distinct geographic areas due to the cryptic behaviour of the species [30]. A total of 34,50,872 reads from the 16S rRNA were retained after the demultiplexing, quality filtering, and chimera removal. The average reads of each sample were 95,857 and ranged from 68004 (minimum) to 111599 (maximum). These reads were assigned to 13650 Amplicon Sequence variants (ASVs). A total of, 1282 ASVs was retained after the removal of singletons (10,154), low variance (143) and low abundance (2071) ASVs. The Venn analysis revealed 208 ASVs were shared by all the four families, while, 670 unique ASVs for Araneidae, 510 for Lycosidae, 484 for Salticidae and 590 for Tetragnathidae were recovered (Fig 1). The maximum number of ASVs (200) were shared between the spider families, Lycosidae and Salticidae while the minimum ASVs i.e. 13 were shared between Araneidae and Salticidae. Furthermore, all rarefaction curves were saturation which clearly indicated that the appropriate sequencing depth has been achieved for the taxonomic classification (S1 Fig). ASVs were assigned to taxonomic groups using the SILVA database with a 99% similarity cut-off. Moreover, the identified ASVs were distributed among 22 bacterial phyla and 150 families.

**Fig 2:**
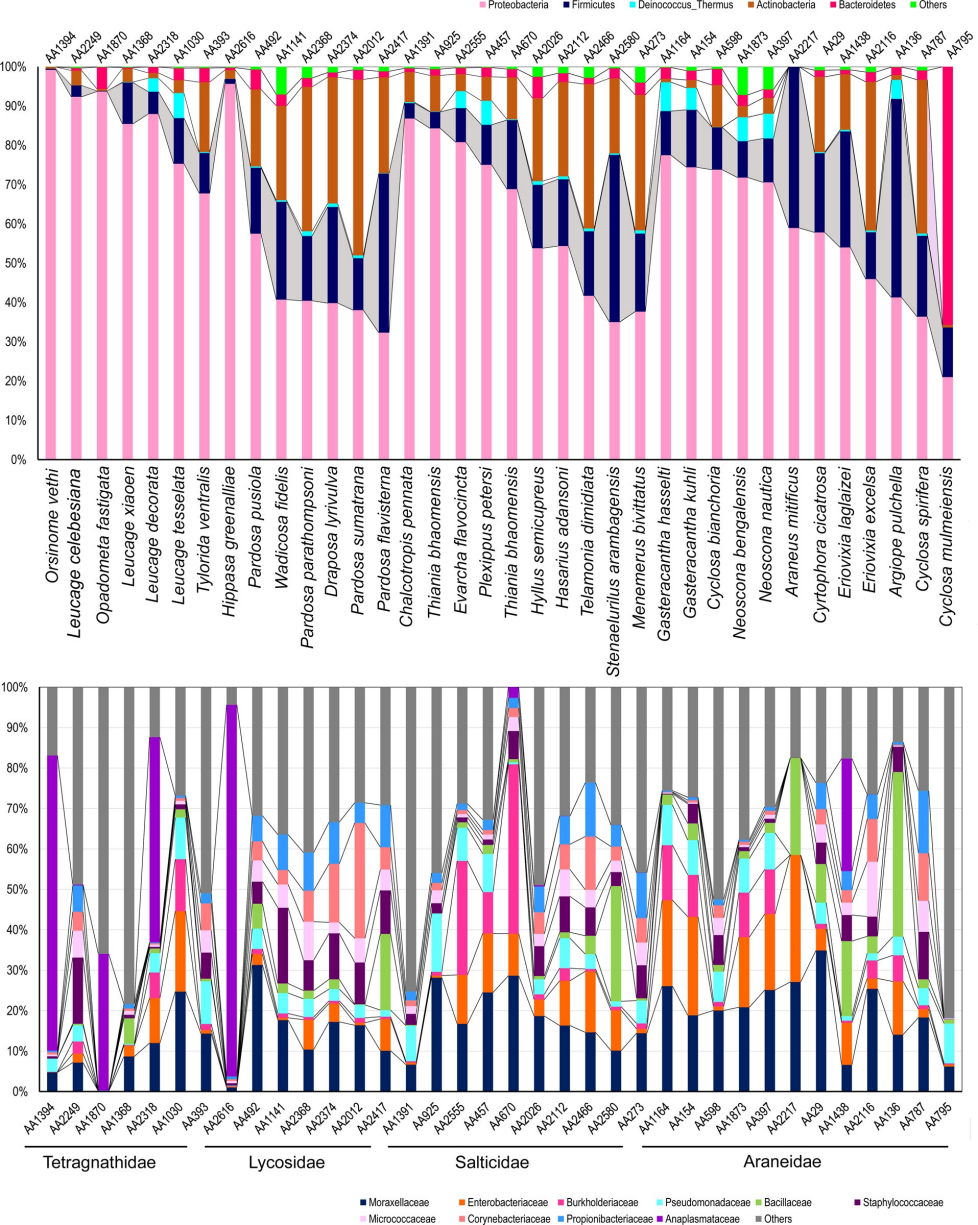
The gut bacterial diversity of spider species based on dominant phyla and families.

The major phyla such as Proteobacteria, Firmicutes, Actinobacteria and Bacteroidetes accounted ~97% of the total bacterial diversity in all the spider species (Fig 2). The most abundant taxa i.e. Proteobacteria, contributed ~57% in Araneidae, 49% in Lycosidae, 61% in Salticidae and 87% in Tetragnathidae followed by Firmicutes (6–20%), Actinobacteria (4–26%), Bacteroidetes (2–4%), Deinococcus _Thermus (1%) etc.

A total of 78 orders were observed in the current dataset. Among them the five major orders that are abundant in the gut of all the four spider species were Pseudomonadales (22%), Bacillales (12%), Enterobacteriales (9%), Rickettsiales (8%), and Micrococcales (5%). Further, the gut diversity of spider species belonging to family Araneidae was represented by five major orders such as Pseudomonadales, Bacillales, Enterobacteriales, Bacteroidales, and Betaproteobacteriales. They together contributed 66% to the total bacterial diversity. On other hand, the spider species belonging to the family Tetragnathidae were represented by Rickettsiales, Pseudomonadales, Rhizobiales, Enterobacteriales, and Bacillales, contributing to 80% of the total bacterial diversity. The gut bacterial diversity in spider species of family Lycosids were represented by Pseudomonadales, Bacillales, Rickettsiales, Corynebacteriales, and Micrococcales, contributed 65% of the total bacterial diversity. The family Salticidae, represented by Pseudomonadales, Bacillales, Enterobacteriales, Micrococcales and Propionibacteriales, contributed 56% to the total bacterial diversity.

The gut diversity of the members of all four spiders families were represented by 150 bacterial families. The bacterial families Moraxellaceae and Enterobacteriaceae were distributed across the four spider families, with the exception of the Lycosidae family where Enterobacteriaceae were not detected (Fig 2). In addition to this, few bacterial families were detected in specific spider family/families i.e. Bacillaceae and Prevotellaceae in Araneidae only; Rickettsiaceae and Rhizobiaceae in Tetragnathidae only; Anaplasmataceae in Tetragnathidae and Lycosidae; Pseudomonadaceae in Araneidae and Salticidae; Propionibacteriaceae in Lycosidae and Salticidae; Staphylococcaceae and Corynebacteriaceae only in Lycosidae; and Burkholderiaceae in Salticidae.

**Fig 3:**
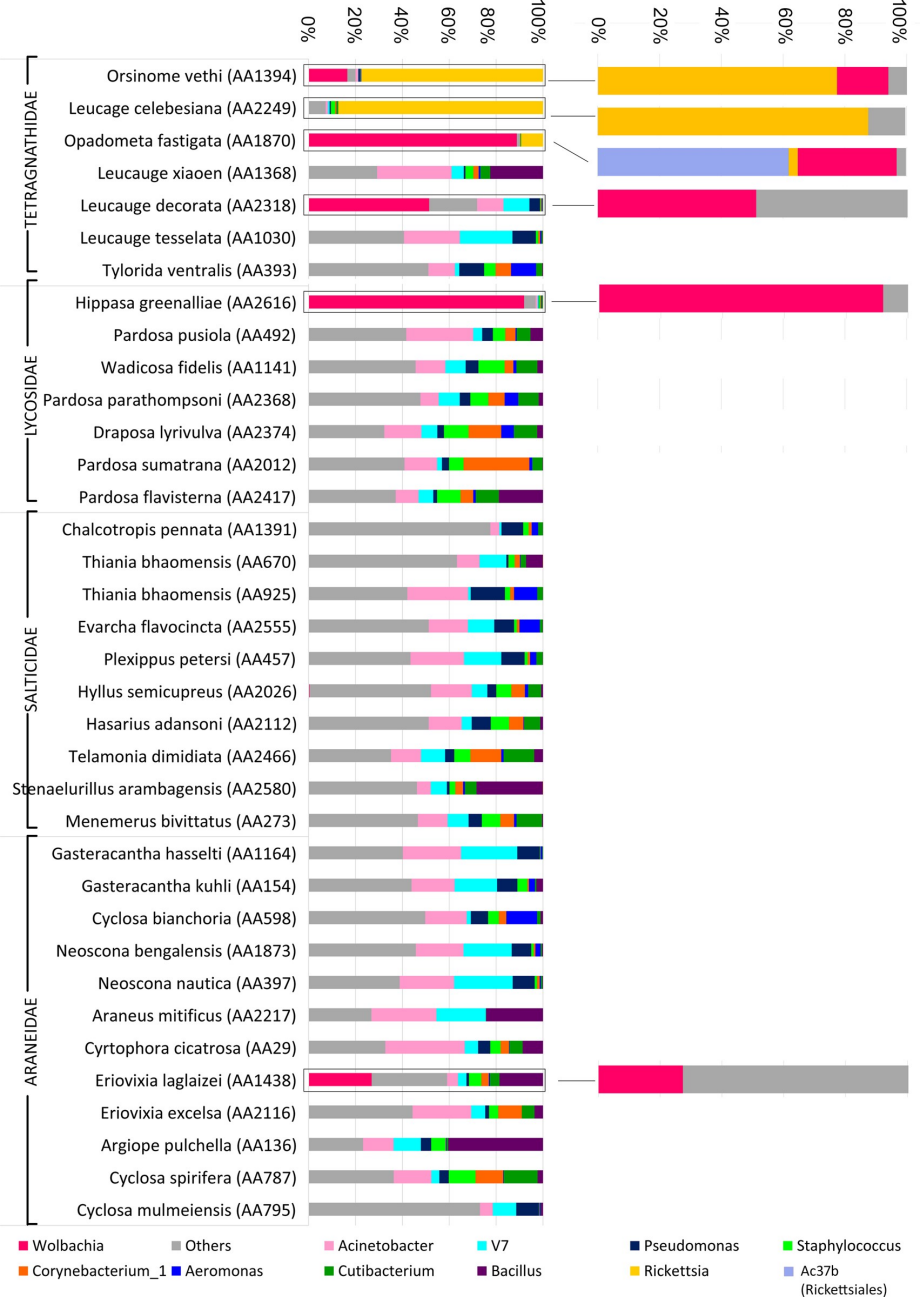
Genus level gut bacterial diversity of spider species along with influence of endosymbiont genera.

A total of 384 bacterial genera was detected. The bacterial genera, *Acinetobacter* (15%), V7clade (9%), *Wolbachia* (8%), *Pseudomonas* (5%), *Bacillus* (6%) were the most abundant in the gut of four spider families. Abundance of the other genera, *Rickettsia* (5%), *Corynebacterium*_1 (4%), *Staphylococcus* (4%), *Cutibacterium* (4%), and *Aeromonas* (2%) was also observed (Fig 3). Furthermore, the genus *Prevotella_9* showed 62% in a single species *Cyclosa mulmeiensis* (Araneidae). The gut bacterial diversity of two populations (AA670 & AA925) of *Thiania bhamoensis* was variable. The abundance of the genus *Acinetobacteria* varies from 10% (AA670) to 26% (AA925); *Pseudomonas* varies from 1% (AA670) to 15% (AA925), while the genus clade V7 varies from 1% (AA925) to 11% (AA670). The genus *Aeromonas* (10%) was found only in AA925 sample, while, the genera *Bacillus* (7%) and *Klebsiella* (27%) were detected in the AA670 sample.

### Endosymbionts abundance

We observed *Rickettsia* and *Wolbachia* as the major endosymbionts in the spider gut. These two bacterial genera have altered the other bacterial abundance in the spider species. The spider species, *Orsinome vethi* was detected with 77% *Rickettsia* and 17% *Wolbachia* and contributed 94% of total bacterial diversity. The spider species, *Opadometa fastigata* was detected with 3% *Rickettsia*, 32% *Wolbachia*, and 62% unidentified strain of the order Rickettsiales (Ac37b), which was 97% of the total bacterial diversity. Whereas, in *Leucage celebesiana* was detected with 88% Rickettsia, and *Leucage decorata* with 51% *Wolbachia*. *Wolbachia* showed an abundance of 92% in *Hippasa greenalliae*, and 27% in *Eriovixia laglazei*. This data indicated that the presence of the genus *Wolbachia* and *Rickettsia* in these species alter the abundance of other bacterial taxa except in *Eriovixia laglazei* (S1 Table and Fig 3).

### Diversity measures

**Fig 4:**
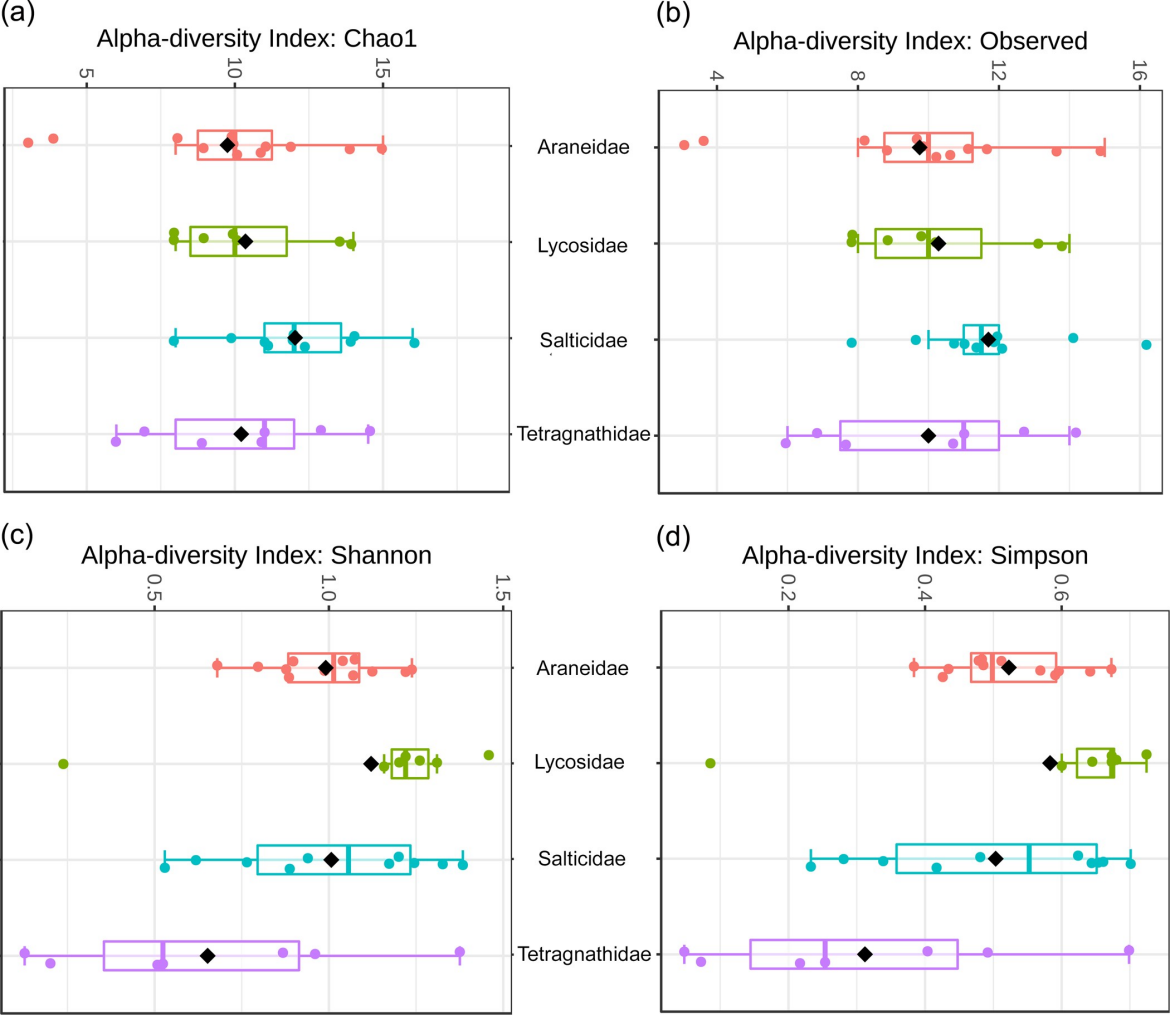
Alpha-diversity of four spider families based on (a) observed, (b) Chao1, (c) Shannon and (d) Simpson metrices.

We used four diversity measures Chao1, Observed, Shannon and Simpson with the ANOVA statistical method to measure the alpha diversity in all four spider families (Fig 4). The alpha diversity of the four spider families ranged from 3–16 (Chao1, Observed), 0.05 to 1.3 (Shannon) and 0.07 to 0.16 (Simpson). The richness of four spider families based on two diversity measures (Shannon and Simpson) was significant (p < 0.05), while Chao1, Observed was non-significant (p> 0.05) (S2 Table). Thus, the community richness for the Lycosidae family was more diverse than that of the other three families.

**Fig 5:**
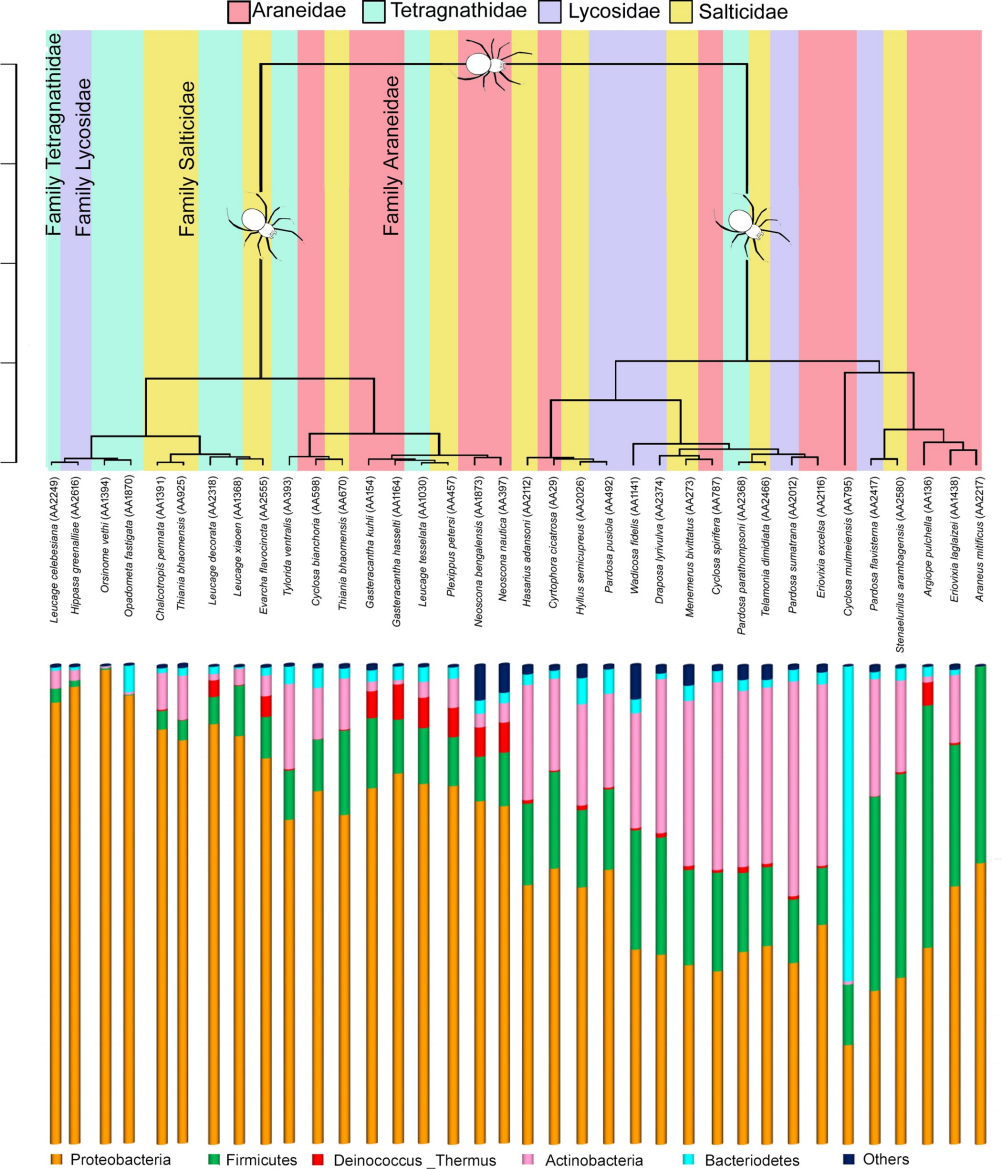
Dendrogram of β-diversity of spider species under 4 families, along with phylum gut bacterial diversity.

The beta diversity in the gut of the four spider families based on NMDS was calculated using the Bray-Curtis index method (S2 Fig) and the dendrogram using the Unweighted Unifrac distance method (Fig 5). Both the methods showed the similar results with stress value of 0.12 (Bray-Curtis index) and 0.13 (Unweighted Unifrac distance). In both the analyses, four spider species showed the distinct differences in their gut bacterial diversity with respect to others. The Araneidae species (*Cyclosa mulmeiensis*, AA795) showed a high abundance of the Bacteroidetes phylum in comparison with other spider species. The Tetragnathidae species, *Orsinome vethi* (AA1394) and *Opadometa fastigata* (AA1840) showed a high abundance of Proteobacteria in compare to other spider species. The Araneidae species, *Araneus mitificus* (AA2217) was detected with only two phyla, i.e. Proteobacteria and Firmicutes.

**Fig 6:**
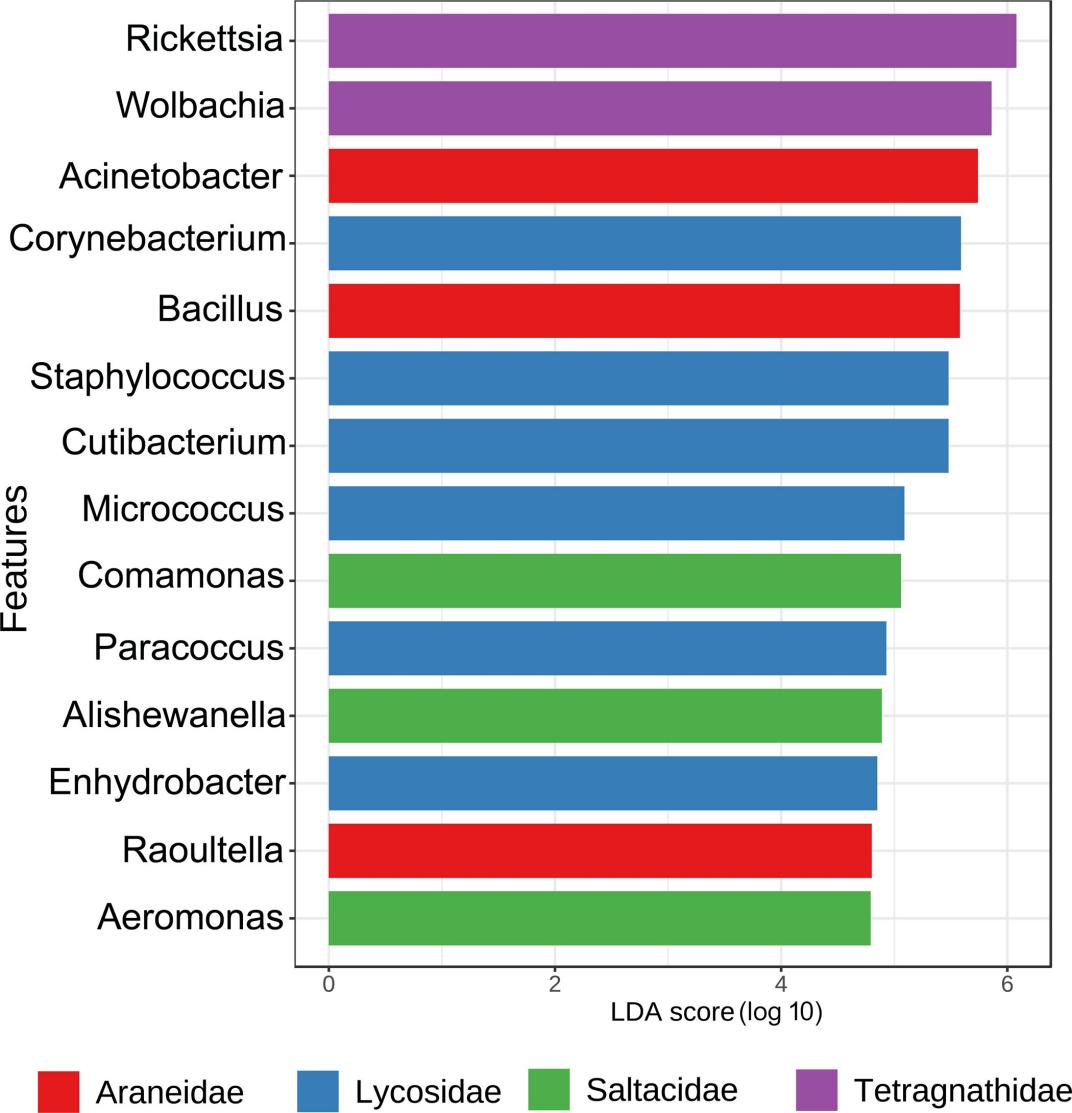
LDA results based on genus level in four spider families.

Linear Discriminant Analysis (LDA) Effect Size (LEfSe) was implemented to examine the significant bacterial diversity between the species of four spider families with cut off 0.2 and Log LDA score 4 parameters. A total 48 significant features were detected, of which 14 are represented (Fig 6). The presence of the genera *Rickettsia* and *Wolbachia* in the family Tetragnathidae was a distinct difference as compared to other spider families. The genera *Acinetobacter*, *Bacillus*, and *Raoultella* significantly explain the differentiation of the bacterial diversity of the Araneidae family in comparison with other spider families. The genera *Corynebacterium*, *Staphyloccocus*, *Cutibacterium*, *Micrococcus*, *Paracoccus*, and *Enhydrobacter* were found in the Lycosidae family and are distinct from other spider families. The genera *Comamonas*, *Alishewanella*, and *Aeromonas* constituted the distinguishing features of the Salticidae family.

### Comparative functional analysis

**Fig 7:**
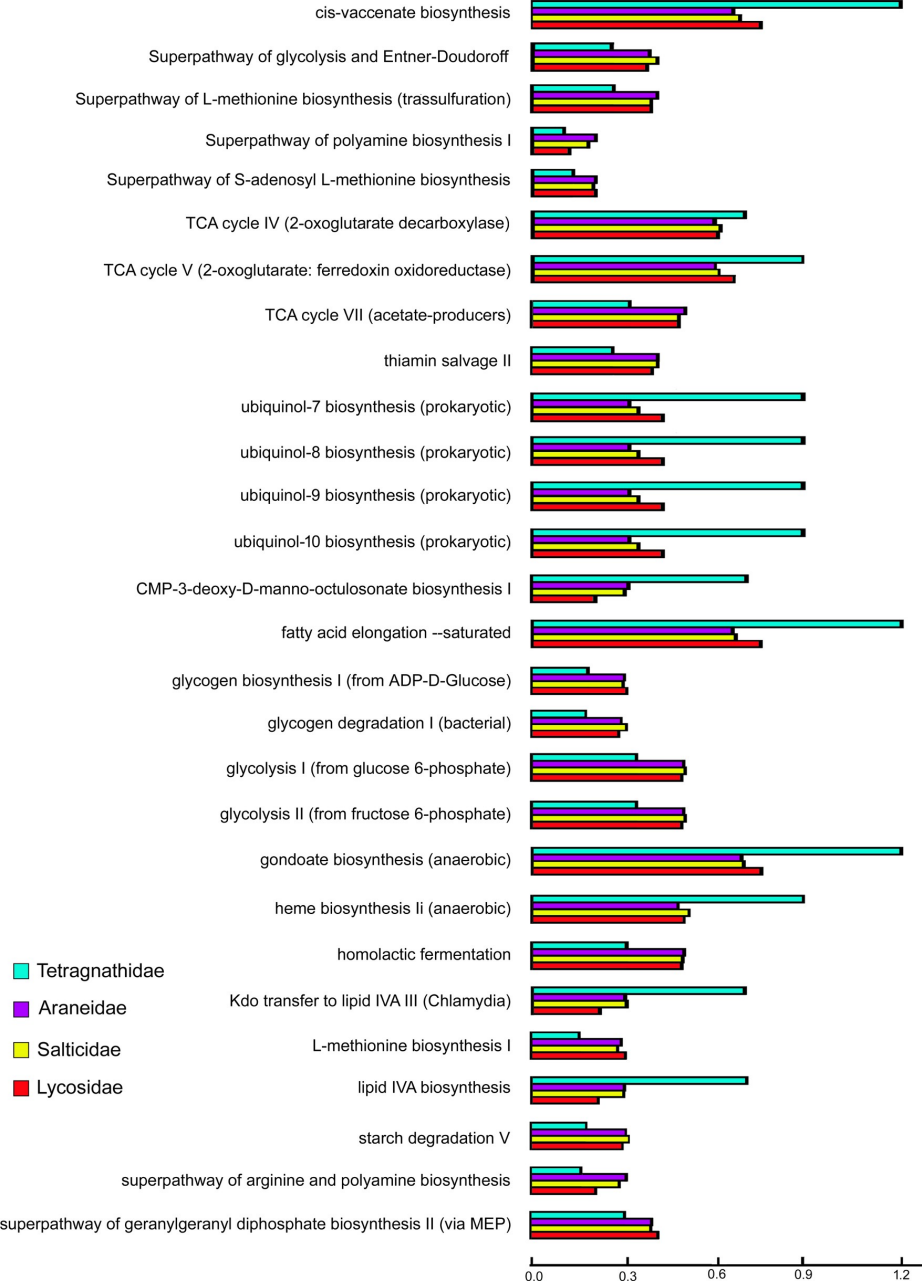
Representation of the common predicted functional metabolic pathway between four spider families.

The predicted functional pathway analysis was carried out using PICRUSt2 (Phylogenetic Investigation of Communities by Reconstruction of Unobserved States) with the KEGG database (Kyoto Encyclopedia of Genes and Genomes). This analysis predicted 159 functional pathways among the four spider families (S3–S8 Figs). Out of the 159 functional pathways, Araneidae shared 116 pathways with Tetragnathidae (S3 Fig), 79 with Lycosidae (S4 Fig) and 13 with Salticidae (S5 Fig). While Lycosidae shared 64 pathways with Tetragnathidae (S6 Fig) and 41 with Salticidae (S7 Fig). Tetragnathidae shared 112 pathways with Salticidae (S8 Fig). Twenty eight (28) pathways were shared among the four spider families (Fig 7). The carbohydrate metabolism, cofactors and vitamin metabolism, amino acids metabolism, organic compound biosynthesis, fatty acid elongation and synthesis, heme biosynthesis and lipid biosynthesis pathways were observed in the all the four families.

## Discussion

It is the largest effort to document the gut bacterial diversity of spiders. We studied 35 species from four families (Araneidae, Lycosidae, Salticidae, and Tetragnathidae) of spiders with their predictive functional metabolic pathways. The bacterial diversity in the gut of four families of spiders was distributed over 22 phyla and 150 families. Our study found that the most dominant phyla in the gut all spider species are Proteobacteria (61.5%) and Firmicutes (16.1%), and contributed to 78.5% of the total gut bacterial diversity. This result is well supported by previous studies and has shown that the most dominant phyla in the gut of spiders are Proteobacteria and Firmicutes [19, 21–23]. Furthermore, the abundance of Proteobacteria as the dominant phylum is well documented in other arthropods also [12] such as scorpions [16], ticks [17], cockroaches [31], and butterflies [15]. Other major phyla like Actinobacteria and Bacteroidetes have also been observed and contribute to 18.4% of total gut bacterial diversity.

Few differences were observed in the composition of the gut microbiome in the species studied. The genera *Acinetobacter*, *Pseudomonas*, *Staphylococcus*, *Corynebacterium*_1, *Cutibacterium*, and *Aeromonas* were detected in the gut of the four families of spiders. Whereas genus *Bacillus* was detected in all three families of spiders, with the exception of the family Tetragnathidae. The genus *Wolbachia* has been detected in Araneidae and Tetragnathidae; *Rickettsia* in Tetragnathidae only; *Prevotella*_9 in Araneidae; *Micrococcus*, *Paracoccus* and *Enhydrobacter* in Lycosidae; and *Alishewanella*, and *Comamonas* in Salticidae. *Acinetobacter*, and *Pseudomonas* of the phylum Proteobacteria are the most dominant taxa in the gut of the spider families as well as in other arthropods [12, 32–34]. The functional analysis revealed that *Acinetobacter* may be responsible for cofactor and vitamin metabolism in the gut of all the spider families as it has been reported to be involved in the biosynthesis of ubiquinol (https://biocyc.org/META/NEW-IMAGE?type=PATHWAY&object=UBISYN-PWY). While the genus *Pseudomonas* was reported to be involved in the organophosphate degradation [35], energy and lipid metabolism [8].

In the present study, genera *Bacillus* and *Staphylococcus* of Phylum Firmicutes have been observed in all the spider families. They may be involved in the degradation of polysaccharides and aromatic compounds [36]. Members of the genus *Staphylococcus* were usually involved in detoxification, and defensive behaviour against natural enemies [37]. *Cutibacterium* in the gut of spiders may be involved in fatty acid metabolism as reported earlier in two-spotted spider mite, *Tetranychus urticae* (https://www.genome.jp/kegg-bin/show_pathway?pad00061). The genera *Corynebacterium*_1 and *Aeromonas* were detected in spiders and dipterans [38]. Earlier studies have revealed that the presence of *Corynebacterium*_1 in the gut of spiders is due to consumption of dipterans as food [39].

Two endosymbiont genera, *Wolbachia* and *Rickettsia* of Phylum Proteobacteria are reported in the in the gut of spiders as well as in other arthropods such as insects [40–43] etc. These two genera are known for their reproductive manipulation ability in arthropods [44]. In the current study, *Wolbachia* was most abundant in 6 species, *Leucage decorata*, *Leucage celebesiana*, *Opadometa fastigata*, and *Orsinome vethi* of family Tetragnathidae, *Hippasa greenalliae* of Lycosidae and *Eriovixia laglazei* of Araneidae. While the gut of the Salticidae family members have no evidence of this genus contrary to earlier studies [45]. This genus is generally responsible for symbiotic associations, ranging from mutualism to parasitism in arthropods [46]. The genus *Rickettsia* has only been reported only in 2 species of the family Tetragnathidae (*Orsinome vethi* and *Opadometa fastigata*). The genus *Prevotella*_9 is reported in only one species of family Araneidae (*Cyclosa mulmeiensis*). This genus has been reported to be responsible for the larva metamorphosis into adults in the bark beetle [32]. The genera *Paracoccus* and *Micrococcus* have been reported in gut of Lycosids, ticks [47] and dipterans [48]. In the present study, two populations of *Thainia bhamoensis* (AA670, AA925) had few differences in the abundance of two genera *Actinobacteria*, and *Pseudomonas*. The genus *Aeromonas* was detected in AA925 and *Klebsiella* in AA670. Gut bacteria of spider families may be involved in carbohydrate metabolism, amino acid metabolism, fatty acids metabolism, cofactors and vitamin metabolism.

## Conclusion

Our results showed that almost all the spider species belonging to four families had a very similar gut bacterial composition and structure. There were few exceptions like: six species (*Hippasa greenalliae*, *Leucage decorata*, *Leucage celebesiana*, *Orsinome vethi*, *Opadometa fastigata*, and *Eriovixia laglazei*) were infected by either *Wolbachia* or *Rickettsia* or both. The presence of these endosymbionts in turn affected the abundance profile of other gut bacteria in these species. Moreover the richness of gut bacterial diversity in the family Lycosidae was much higher than in Salticidae, Tetragnathidae and Araneidae. Furthermore, in order to understand the profile of microbial diversity and the relationship between host and habitat in spiders, extensive sampling and study of the gut microbiome is necessary.

## Material and methods

The specimens were collected from the six Indian states of Assam, Chhattisgarh, Karnataka, Kerala, Odisha and West Bengal (Table 1). We used two methods of collection: by hand picking method under the wood logs, foliage of leaves, and stones; and by sweep net collection from the vegetation. Each specimen was placed in a separate 15 ml tube. After three to four hours, each specimen was transferred to 100% alcohol to be stored at -20°C. All specimens were morphologically identified using published literature and available taxonomic keys. The species included in this study are not endangered or protected.

**Table 1:** Collection details of the Species of four spider families with their sample code.

S. No	Sample Id	Species	Family	Locality	Latitude	Longitude
1.	AA2217	*Araneus mitificus*	Araneidae	West Bengal	25.01 N	88.14 E
2.	AA136	*Argiope pulchella*	Araneidae	West Bengal	23.84 N	87.61 E
3.	AA598	*Cyclosa bianchoria*	Araneidae	Andhra Pradesh	16.50 N	80.64 E
4.	AA795	*Cyclosa mulmeiensis*	Araneidae	West Bengal	23.23 N	87.86 E
5.	AA787	*Cyclosa spirifera*	Araneidae	West Bengal	24.22 N	88.24 E
6.	AA29	*Cyrtophora cicatrosa*	Araneidae	West Bengal	22.56 N	88.44 E
7.	AA2116	*Eriovixia excelsa*	Araneidae	West Bengal	22.56 N	88.44 E
8.	AA1438	*Eriovixia laglaizei*	Araneidae	West Bengal	22.50 N	88.33 E
9.	AA1164	*Gasteracantha hasselti*	Araneidae	West Bengal	22.96 N	77.56 E
10.	AA154	*Gasteracantha kuhli*	Araneidae	West Bengal	22.54 N	88.36 E
11.	AA1873	*Neoscona bengalensis*	Araneidae	West Bengal	22.54 N	88.39 E
12.	AA397	*Neoscona nautica*	Araneidae	West Bengal	22.85 N	88.56 E
13.	AA2374	*Draposa lyrivulva*	Lycosidae	Odisha	19.26 N	84.86 E
14.	AA2616	*Hippasa greenalliae*	Lycosidae	Odisha	20.27 N	85.80 E
15.	AA2417	*Pardosa flavisterna*	Lycosidae	Odisha	20.27 N	85.80 E
16.	AA2368	*Pardosa parathompsoni*	Lycosidae	Odisha	19.58 N	84.68 E
17.	AA492	*Pardosa pusiola*	Lycosidae	Assam	26.73 N	94.15 E
18.	AA2012	*Parrdosa sumatrana*	Lycosidae	Assam	26.73 N	94.15 E
19.	AA1141	*Wadicosa fidelis*	Lycosidae	Kerala	11.59 N	75.77 E
20.	AA1391	*Chalcotropis pennata*	Salticidae	Kerala	11.59 N	75.77 E
21.	AA2555	*Evarcha flavocincta*	Salticidae	Odisha	20.22 N	85.80 E
22.	AA2112	*Hasarius adansoni*	Salticidae	Odisha	20.22 N	85.80 E
23.	AA2026	*Hyllus semicupreus*	Salticidae	Odisha	19.58 N	84.68 E
24.	AA273	*Menemerus bivittatus*	Salticidae	Assam	26.73 N	94.15 E
25.	AA457	*Plexippus petersi*	Salticidae	Chhattisgarh	22.09 N	81.25 E
26.	AA2466	*Telamonia dimidiata*	Salticidae	Odisha	19.26 N	84.86 E
27.	AA925	*Thiania bhaomensis*	Salticidae	Assam	27.59 N	95.68 E
28.	AA670	*Thiania bhaomensis*	Salticidae	Assam	26.68 N	92.81 E
29.	AA2580	*Stenaelurillus arambagensis*	Salticidae	Odisha	19.36 N	84.91 E
30.	AA2249	*Leucauge celebesiana*	Tetraganthidae	Odisha	19.32 N	84.88 E
31.	AA2318	*Leucauge decorata*	Tetraganthidae	Odisha	19.26 N	84.86 E
32.	AA1030	*Leucauge tesselata*	Tetraganthidae	Assam	26.73 N	94.15 E
33.	AA1368	*Leucauge xiaoen*	Tetraganthidae	Karnataka	13.10 N	77.85 E
34.	AA1870	*Opadometa fastigata*	Tetraganthidae	Odisha	19.26 N	84.86 E
35.	AA1394	*Orsinome vethi*	Tetraganthidae	Kerala	11.59 N	75.77 E
36.	AA393	*Tylorida ventralis*	Tetraganthidae	Assam	26.73 N	94.15 E

### DNA isolation and 16srRNA amplicon sequencing

Before dissection, each specimen was washed three times with Milli-Q water to remove the environmental contamination. After washing, the abdomen of ten specimens of each species was removed and pooled to isolate the DNA. The DNA was isolated from pooled specimens of each species using a DNeasy Blood & Tissue Kit (Qiagen) as per manufacturer protocol. The quantity and quality of the extracted DNA was quantified by Qubit 2.0 Fluorometer (Q32866, Thermofisher) and by agarose gel electrophoresis (Cell BioScience Alphalmager MINI). PCR reaction (Polymerase Chain Reaction) was carried out for the amplification of 36 extracted DNA of 35 spider species (one species has two geographical replicates) using primer sets of V3-V4 region of 16srRNA. The reaction mixture (25 μL) includes both forward and reverse primer (1 μl of each), Taq DNA polymerase (0.5 μl), dNTPs (1 μl), 10 × buffer (2.5 μl), DNA template, Milli-Q water. The reaction cycle involved the following steps: 5 min at 98°C followed by 35 cycles for 30 s at 98°C (denaturation), 45 s at 53°C (annealing), and 72°C for 45 s (elongation), and 7 min at 72°C (final extension). The PCR products were visualized by agarose gels. The sequencing of V3-V4 region of 16srRNA was carried out on the Illumina MiSeq Platform. The raw reads were submitted under the BioProject ID PRJNA638522 with accession number SAMN15196477 to SAMN15196488, SAMN15580727 to SAMN15580750 to The National Center for Biotechnology Information (NCBI) GenBank Portal.

### Bioinformatics and statistical analyses

The paired end raw reads (4441444) of 36 specimens from 35 species with minimum (121255) and maximum (125368) were generated. These reads were merged into single reads in QIIME2 (ver. 2019.10) using demultiplexing [49]. These single reads were quality filtered, trimmed, de-noised and merged by using a DADA2 pipeline in QIIME2 [50]. The chimeric reads were identified and removed and non-chimeric reads were grouped and assigned to Amplicon Sequence Variants (ASVs). QIIME2 q2-feature-classifier plugin was used for the taxonomic classification of these ASVs based on 99% similarity on SILVA database (version 132). The taxonomy file and feature tables were generated for the downstream analysis.

Further analysis was carried out on The MicrobiomeAanalyst (web based tool), using Marker Data Profiling (MDP) and recovered 13650 ASVs [51]. These ASVs were again subjected to singleton removal, low variance and low abundance features with a final recovery of 1282 ASVs. The unfiltered data was used to calculate the diversity measures. The Observed, Chao1, Shannon and Simpson diversity measures with T-test/ANOVA statistical methods were used for alpha diversity to calculate the diversity and richness. The PERMANOVA based statistical method for Bray Curtis and Ward’s linkage based method for unweighted UniFrac distance measure were used for beta diversity to calculate the similarity of gut bacterial diversity in spider species. Nonmetric Multidimensional Scaling (NMDS) and Dendrogram were used for beta diversity analysis. We have used linear discriminant analysis (LDA) of effect size with cut off 0.2 and Log LDA score 4 parameters to discover biomarkers that differed among four spider families. Colour was used to indicate the different spider families.

A web based tool jvenn was used to view the unique and shared ASVs between the four spider families [52]. To predict the functional metabolic pathways between spider families, The Phylogenetic Investigation of Communities by Reconstruction of Unobserved States (PICRUSt2) [53] based on Kyoto Encyclopedia of Genes and Genomes (KEGG) database was used [54–56]. We used Stamp software [57] for the plotting of predicted metabolic pathway between the four spider families.

## Supporting information

S1 Fig Rarefaction curve of spider species belonging to four spider families. (TIF)

S2 Fig NMDS plot based on Bray-Curtis index of spider species. (TIF)

S3 Fig Representation of the predicted functional metabolic pathway between Araneidae and Tetragnathidae. (TIF)

S4 Fig Representation of the predicted functional metabolic pathway between Araneidae and Lycosidae. (TIF)

S5 Fig Representation of the predicted functional metabolic pathway between Araneidae and Salticidae. (TIF)

S6 Fig Representation of the predicted functional metabolic pathway between Lycosidae and Tetragnathidae. (TIF)

S7 Fig Representation of the predicted functional metabolic pathway between Lycosidae and Salticidae. (TIF)

S8 Fig Representation of the predicted functional metabolic pathway between Salticidae and Tetragnathidae. (TIF)

S1 Table The abundance of the endosymbiont genera of the order Rickettisiales in spider species. (DOCX)

S2 Table Diversity metrics across spider species. (DOCX)
