# Supplementary figures and images for: Interspecific variation and functional traits of the gut microbiome in spiders from the wild: The largest effort so far
Source: PLoS One. 2021 Jul 21;16(7):e0251790. doi: 10.1371/journal.pone.0251790 (PMC8294503; doi:10.1371/journal.pone.0251790)

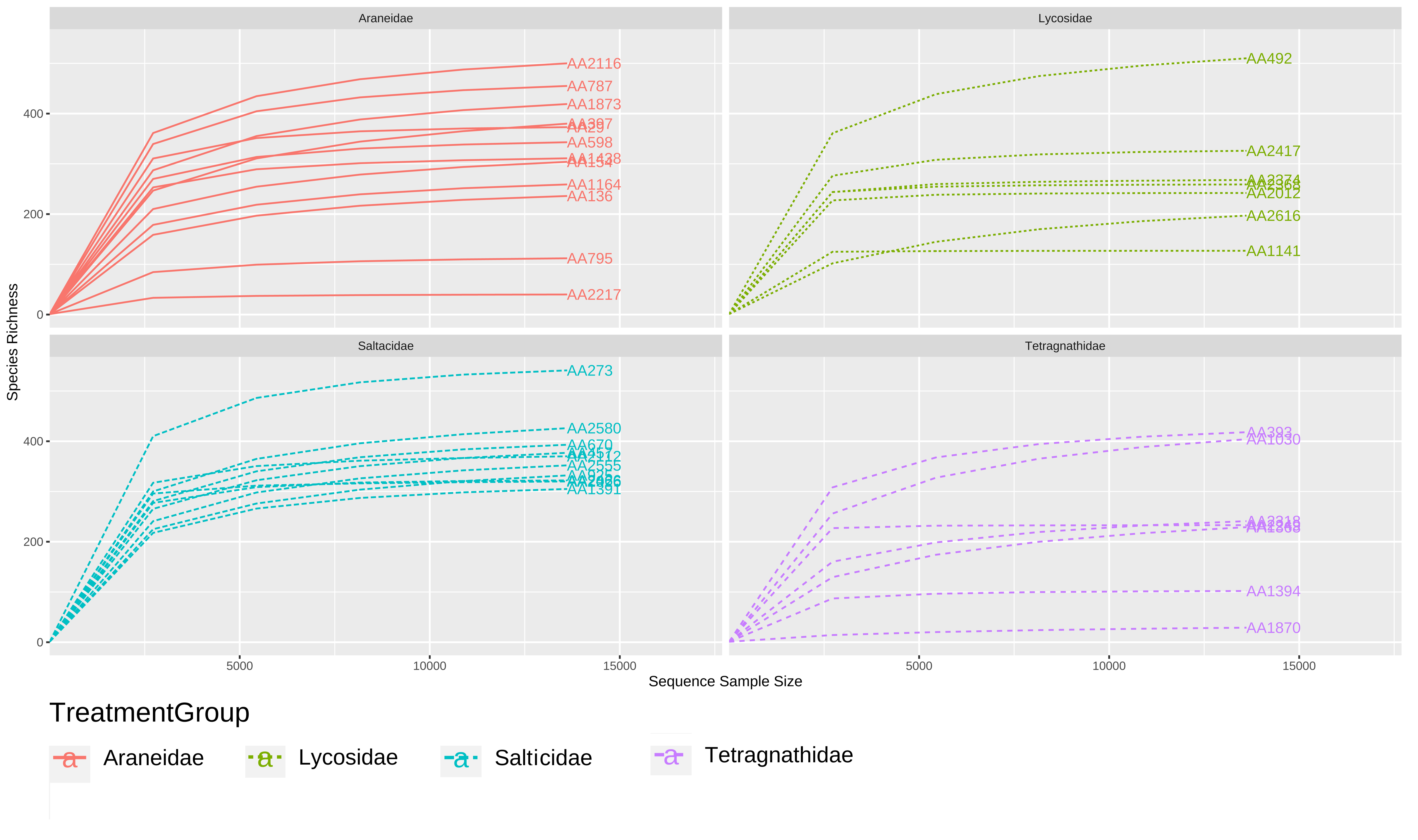

Supplement: S1 Fig — (TIF) [file pone.0251790.s001.tif]

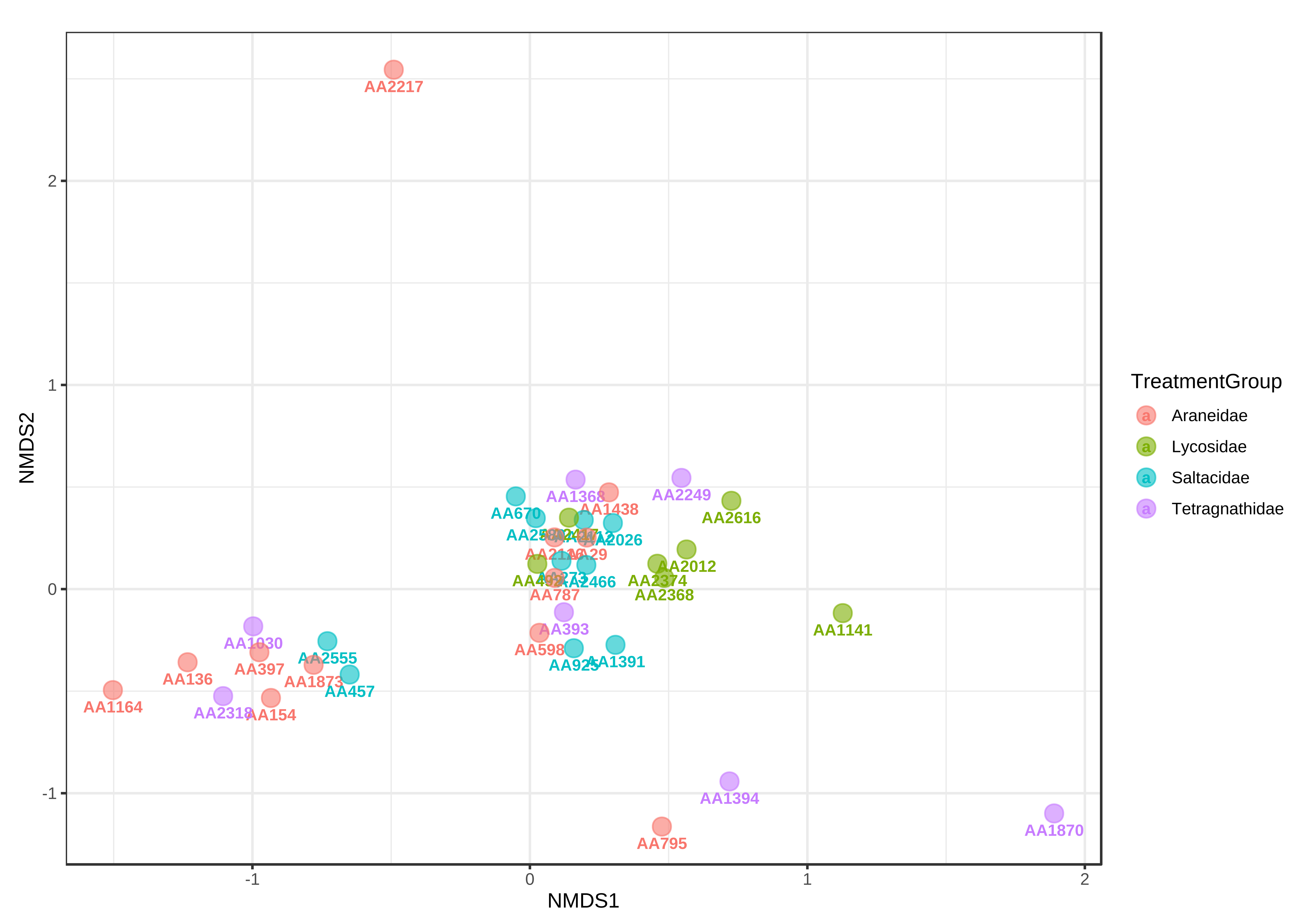

Supplement: S2 Fig — (TIF) [file pone.0251790.s002.tif]

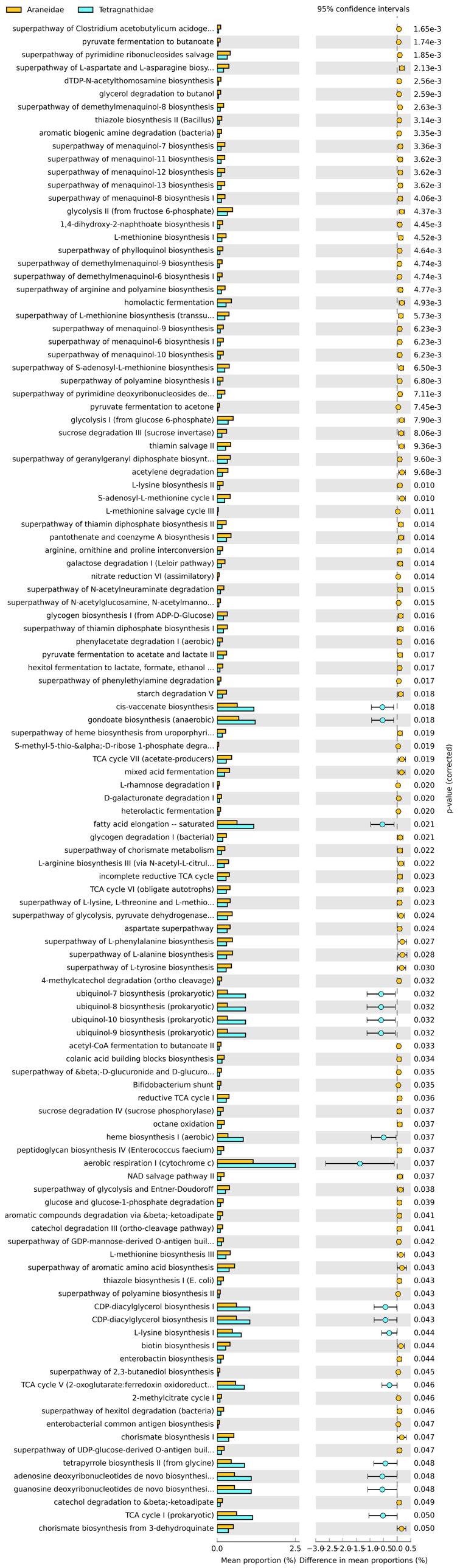

Supplement: S3 Fig — (TIF) [file pone.0251790.s003.tif]

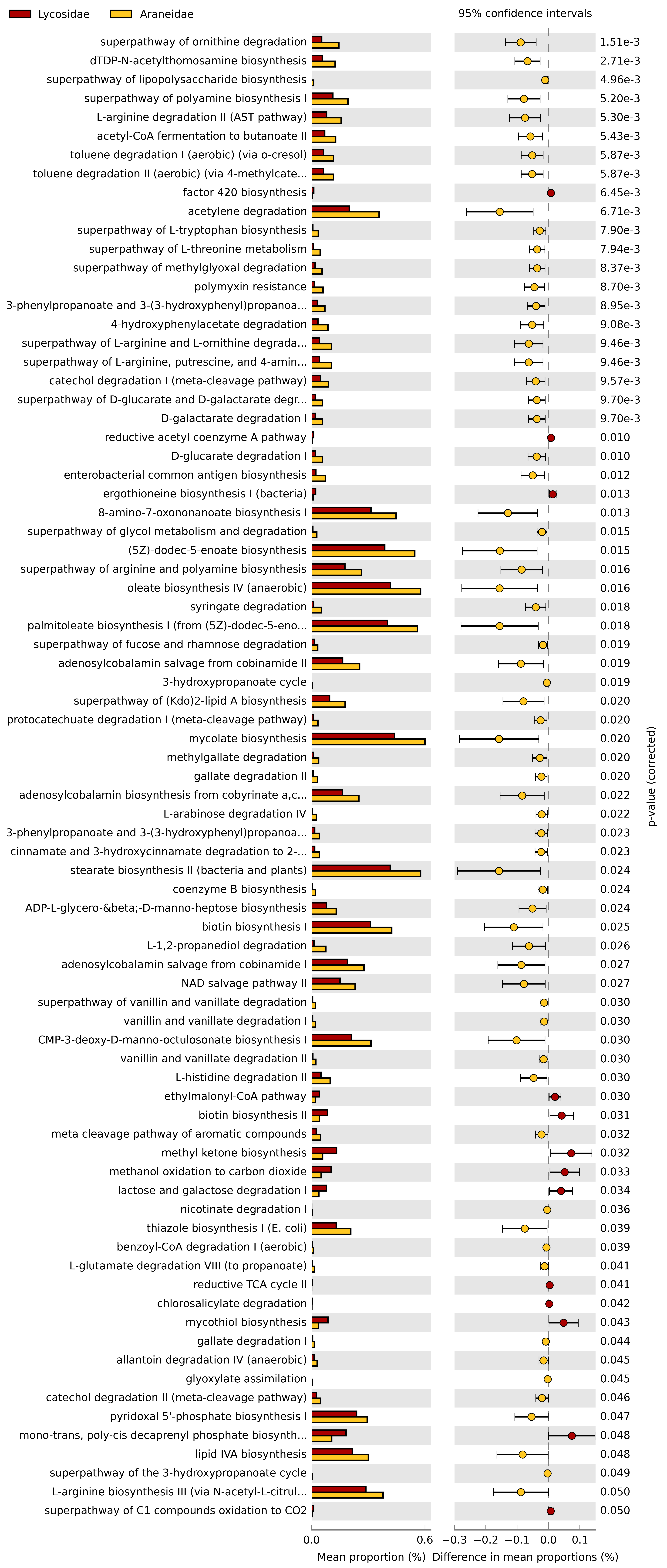

Supplement: S4 Fig — (TIF) [file pone.0251790.s004.tif]

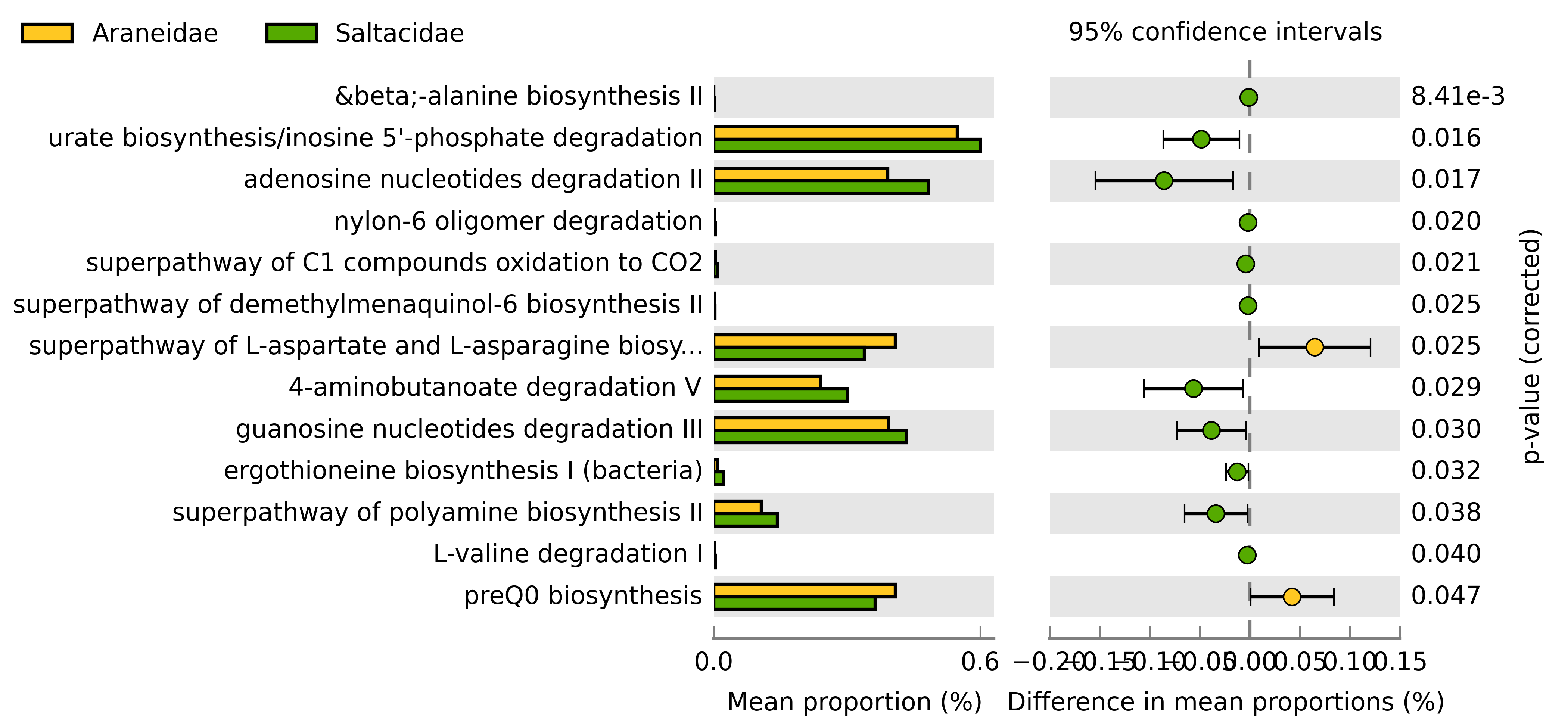

Supplement: S5 Fig — (TIF) [file pone.0251790.s005.tif]

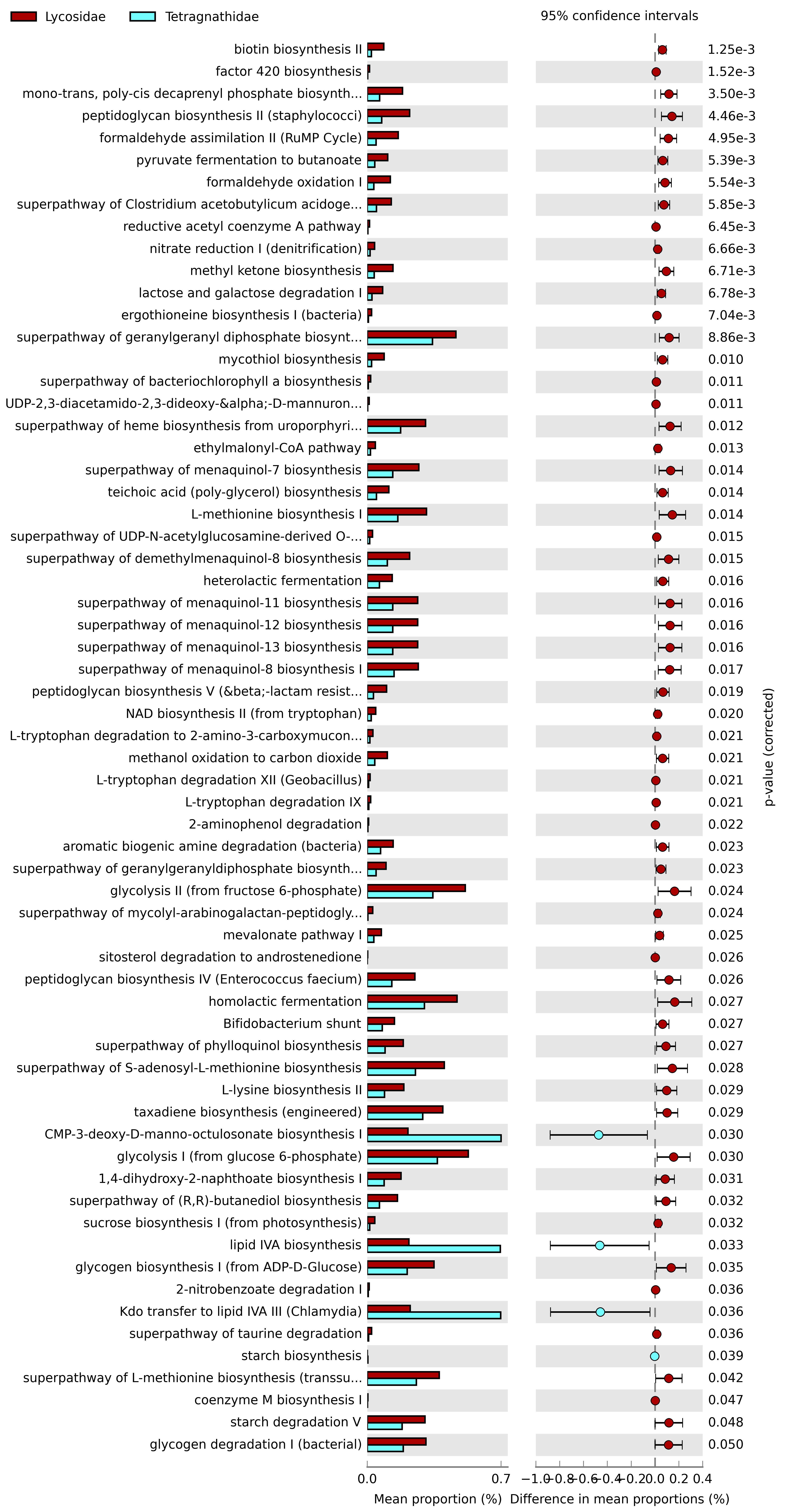

Supplement: S6 Fig — (TIF) [file pone.0251790.s006.tif]

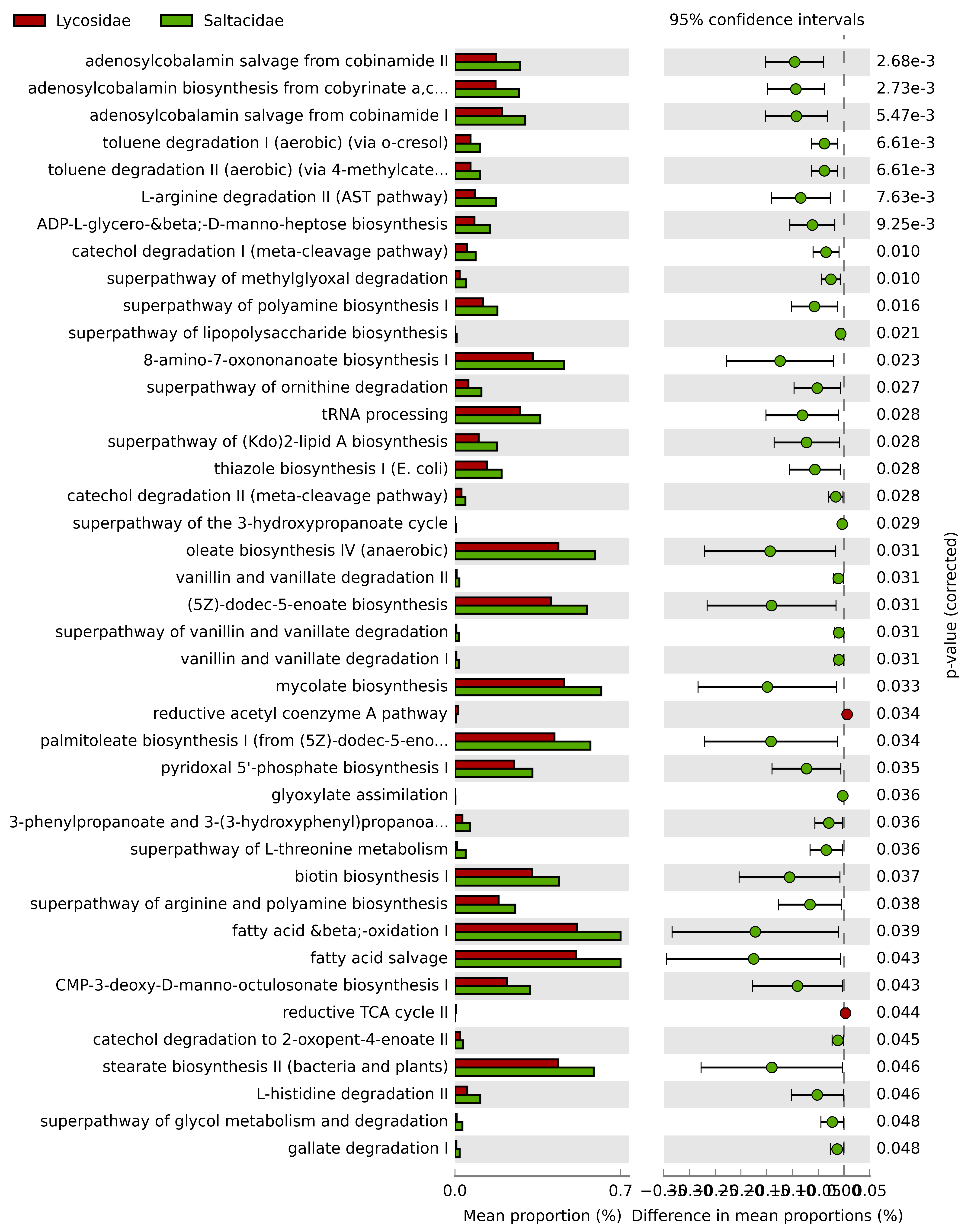

Supplement: S7 Fig — (TIF) [file pone.0251790.s007.tif]

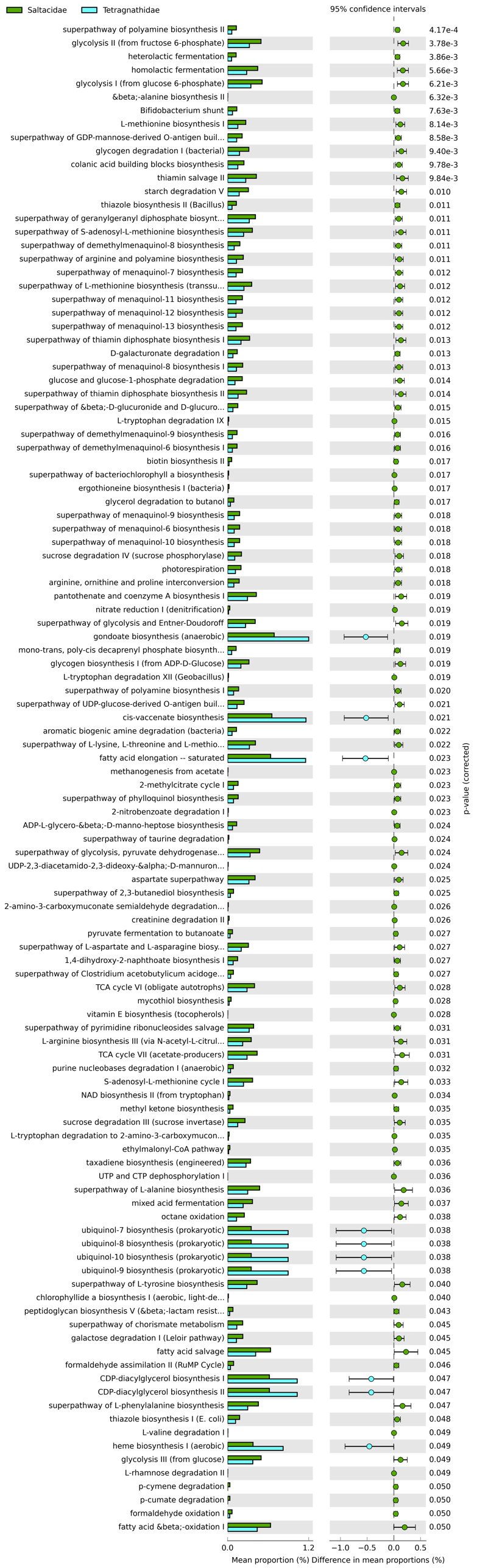

Supplement: S8 Fig — (TIF) [file pone.0251790.s008.tif]
