# Supplementary material for: Interspecific variation and functional traits of the gut microbiome in spiders from the wild: The largest effort so far
Source: PLoS One. 2021 Jul 21;16(7):e0251790. doi: 10.1371/journal.pone.0251790 (PMC8294503; doi:10.1371/journal.pone.0251790)
Supplement: S1 Table — (DOCX) [file pone.0251790.s009.docx]

| Genus | Tetragnathidae | | | | Lycosidae | Araneidae |
| --- | --- | --- | --- | --- | --- | --- |
|  | *Orsinome vethi* (AA1394) | *Leucauge celebesiana* (AA2249) | *Opadometa fastigata* (AA1870) | *Leucauge decorata* (AA2318) | *Hippasa greenalliae* (AA2616) | *Eriovixia laglaizei* (AA1438) |
| *Rickettsia* | 77% | 88% | 3% | 0% | 0% | 0% |
| *Wolbachia* | 17% | 0% | 32% | 51% | 92% | 27% |
| Ac37b (Rickettsiales) | 0% | 0% | 62% | 0% | 0% | 0% |
| Others | 6% | 12% | 3% | 49% | 8% | 73% |
