# Supplementary material for: Interspecific variation and functional traits of the gut microbiome in spiders from the wild: The largest effort so far
Source: PLoS One. 2021 Jul 21;16(7):e0251790. doi: 10.1371/journal.pone.0251790 (PMC8294503; doi:10.1371/journal.pone.0251790)
Supplement: S2 Table — (DOCX) [file pone.0251790.s010.docx]

|  | Family | Species | Sample code | Chao1 | Observed | Shannon | Simpson |
| --- | --- | --- | --- | --- | --- | --- | --- |
|  | Araneidae | *Araneus mitificus* | AA2217 | 44 | 44 | 2.3 | 0.85 |
|  |  | *Argiope pulchella* | AA136 | 265 | 264 | 3.0 | 0.83 |
|  |  | *Cyclosa bianchoria* | AA598 | 348 | 348 | 4.4 | 0.97 |
|  |  | *Cyclosa mulmeiensis* | AA795 | 120 | 119 | 3.0 | 0.93 |
|  |  | *Cyclosa spirifera* | AA787 | 493 | 493 | 4.7 | 0.96 |
|  |  | *Cyrtophora cicatrosa* | AA29 | 382 | 382 | 4.6 | 0.97 |
|  |  | *Eriovixia excelsa* | AA2116 | 523 | 522 | 4.8 | 0.97 |
|  |  | *Eriovixia laglaizei* | AA1438 | 311 | 311 | 3.6 | 0.88 |
|  |  | *Gasteracantha kuhli* | AA154 | 348 | 346 | 4.2 | 0.97 |
|  |  | *Gasteracantha hasselti* | AA1164 | 288 | 288 | 3.8 | 0.95 |
|  |  | *Neoscona bengalensis* | AA1873 | 449 | 448 | 4.4 | 0.97 |
|  |  | *Neoscona nautica* | AA397 | 422 | 421 | 4.1 | 0.96 |
|  | Lycosidae | *Draposa lyrivulva* | AA2374 | 270 | 270 | 4.6 | 0.97 |
|  |  | *Hippasa greenalliae* | AA2616 | 234 | 233 | 0.64 | 0.15 |
|  |  | *Pardosa flavisterna* | AA2417 | 334 | 334 | 4.3 | 0.94 |
|  |  | *Pardosa parathompsoni* | AA2368 | 260 | 260 | 4.7 | 0.98 |
|  |  | *Pardosa pusiola* | AA492 | 543 | 543 | 4.6 | 0.96 |
|  |  | *Parrdosa sumatrana* | AA2012 | 243 | 243 | 4.3 | 0.96 |
|  |  | *Wadicosa fidelis* | AA1141 | 129 | 129 | 4.3 | 0.97 |
|  | Salticidae | *Chalcotropis pennata* | AA1391 | 316 | 316 | 3.6 | 0.92 |
|  |  | *Hyllus semicupreus* | AA2026 | 324 | 323 | 4.9 | 0.98 |
|  |  | *Hasarius adansoni* | AA2112 | 378 | 378 | 4.8 | 0.97 |
|  |  | *Telamonia dimidiata* | AA2466 | 329 | 329 | 4.6 | 0.97 |
|  |  | *Evarcha flavocincta* | AA2555 | 392 | 390 | 4.1 | 0.95 |
|  |  | *Stenaelurillus arambagensis* | AA2580 | 436 | 435 | 4.0 | 0.91 |
|  |  | *Menemerus bivittatus* | AA273 | 544 | 543 | 5.0 | 0.98 |
|  |  | *Thiania bhaomensis* | AA670 | 406 | 406 | 4.2 | 0.95 |
|  |  | *Plexippus petersi* | AA457 | 396 | 396 | 4.4 | 0.97 |
|  |  | *Thiania bhaomensis* | AA925 | 372 | 371 | 4.1 | 0.96 |
|  | Tetragnathidae | *Tylorida ventralis* | AA393 | 427 | 427 | 4.7 | 0.97 |
|  |  | *Leucauge tesselata* | AA1030 | 451 | 451 | 4.1 | 0.96 |
|  |  | *Leucauge xiaoen* | AA1368 | 281 | 279 | 1.5 | 0.47 |
|  |  | *Orsinome vethi* | AA1394 | 99 | 99 | 0.88 | 0.37 |
|  |  | *Opadometa fastigata* | AA1870 | 31 | 31 | 0.90 | 0.51 |
|  |  | *Leucauge celebesiana* | AA2249 | 234 | 234 | 0.99 | 0.23 |
|  |  | *Leucauge decorata* | AA2318 | 275 | 272 | 2.6 | 0.72 |
